# Combination gefitinib and methotrexate treatment for non-tubal ectopic pregnancies: a case series

**DOI:** 10.1093/humrep/deu091

**Published:** 2014-05-07

**Authors:** A.W. Horne, M.M. Skubisz, S. Tong, W.C. Duncan, P. Neil, E.M. Wallace, T.G. Johns

**Affiliations:** 1 MRC Centre for Reproductive Health, The Queen's Medical Research Institute, Edinburgh EH16 4TJ, UK; 2 Translational Obstetrics Group, University of Melbourne, Mercy Hospital for Women, Heidelberg, VIC 3084, Australia; 3 The Ritchie Centre, Monash Institute of Medical Research, Monash University, Clayton, VIC 3168, Australia; 4 Centre for Cancer Research, Monash Institute of Medical Research, Monash University, Clayton, VIC 3168, Australia

**Keywords:** ectopic pregnancy, epidermal growth factor receptor, gefitinib, methotrexate, non-tubal

## Abstract

Non-tubal ectopic pregnancies are a rare subgroup of ectopic pregnancies implanted at sites other than the Fallopian tube. Mortality from non-tubal ectopic pregnancies is higher compared with that for tubal ectopic pregnancies, and they are becoming more common, partly due to the rising incidence of Caesarean sections and use of assisted reproductive technologies. Non-tubal ectopic pregnancies can be especially difficult to treat. Surgical treatment is complex, and follow-up after medical treatment is usually protracted. There is therefore a need for more effective medical therapies to resolve non-tubal ectopic pregnancies and reduce operative intervention. We have recently reported successful use of combination gefitinib (an orally available epidermal growth factor receptor inhibitor) and methotrexate for treatment of tubal pregnancies. To our knowledge, this combination has not been used to treat non-tubal pregnancies. Here we report the use of combination gefitinib and methotrexate to treat eight women with stable, non-tubal ectopic pregnancies at two tertiary academic teaching hospitals (Edinburgh, UK and Melbourne, Australia); five interstitial and three Caesarean section scar ectopic pregnancies. Pretreatment serum hCG levels ranged from 2458 to 48 550 IU/l, and six women had pretreatment hCG levels >5000 IU/l. The women were co-administered 1–2 doses of i.m. methotrexate (50 mg/m^2^ on Day 1, ± Day 4 or Day 7) with seven once daily doses of oral gefitinib (250 mg). The women were monitored until complete resolution of the ectopic pregnancy, defined as a serum hCG <15 IU/l. Time to resolution (days from first methotrexate dose until serum hCG <15 IU/l), safety and tolerability, complication rates and subsequent fertility outcomes were also recorded. All eight women were successfully treated with combination gefitinib and methotrexate. The most common side effects were transient acne/rash and diarrhoea, known side effects of gefitinib. All women promptly resumed menstruation and importantly, three women subsequently conceived spontaneously. Two have delivered a healthy infant at term and the third is currently in her second trimester of pregnancy. Hence, our case series supports a future clinical trial to determine the efficacy of combination gefitinib and methotrexate to treat non-tubal ectopic pregnancies.

## Introduction

Ectopic pregnancies (EPs) have an incidence of ∼1–2% of all pregnancies. They occur when a fertilized ovum implants away from the endometrial cavity, most commonly (>95%) in one of the Fallopian tubes (Jurkovic and Wilkinson, 2011; Fylstra, 2012). EPs can, however, implant in more unusual locations such as within a Caesarean section scar, within the interstitial portion of the Fallopian tubes, in the cervix, on the ovary and potentially anywhere in the abdominal cavity. Mortality from non-tubal ectopic pregnancies is higher than ectopic pregnancies generally, and they are becoming more common due to assisted reproductive technologies, and possibly due to increasing Caesarean section rates (Chetty and Elson, 2009; Verma *et al.*, 2011). Non-tubal ectopic pregnancies are generally difficult to treat and often require a combination of surgical and medical methods.

The literature around management of non-tubal EPs is limited to case reports and series, describing a range of minimally invasive surgical, radiological and medical interventions including laparoscopic and hysteroscopic resection, uterine artery embolization, ultrasound guided injections of the gestational sac with potassium chloride and/or methotrexate, and systemic treatment with drugs such as methotrexate, mifepristone and misoprostil (Verma *et al.*, 2011; Fylstra, 2012). Treatment choice depends on the site, size and pretreatment serum hCG level of the non-tubal EP. In particular, interstitial, Caesarean section scar and cervical EPs often still require surgical resection and/or instrumentation of the uterus, with potential risks to the woman's subsequent reproductive capacity. Because of their rarity as a clinical entity, the best management of non-tubal EPs has been difficult to establish.

In preclinical studies and a phase I single arm, open label study, we demonstrated that co-administering gefitinib (an epidermal growth factor receptor inhibitor) with methotrexate to treat ectopic pregnancies appeared safe. Furthermore, we obtained preliminary data suggesting this combination may have a time to resolution which is 34% faster compared with treatment using methotrexate alone (Nilsson *et al.*, 2013; Skubisz *et al.*, 2013). This suggested adding gefitinib to methotrexate may improve on its efficacy in medically resolving ectopic pregnancies. We therefore wondered whether this combination could be potentially used to treat non-tubal ectopic pregnancies more effectively. Here we report a case series of eight women with non-tubal EPs treated with gefitinib and methotrexate.

## Methods

Institutional human research ethics approval was sought and obtained at both participating sites (Southern Health Human Research Ethics Committee B, 11180B, and Scotland A Research Ethics Committee, 11/AL/0350) to allow administration of combination gefitinib and methotrexate to eight women with non-tubal EPs, and written informed consent was obtained from each participant. The diagnosis of non-tubal EP was made according to set ultrasound diagnostic criteria (Jurkovic *et al.*, 2003; Jurkovic, 2007) in combination with quantitative serum hCG measurement. Inclusion criteria required the women to be assessed as haemodynamically stable (with no pallor, postural change in blood pressure, syncope or pre-syncope, severe abdominal pain or signs of abdominal peritonism, as well as requiring a normal serum haemoglobin and haematocrit) and to have normal baseline white cell count, renal and hepatic indices. Exclusion criteria included severe dermatological, gastrointestinal and pulmonary comorbidities (systems most likely to be affected by combination treatment), allergy to gefitinib and/or methotrexate and Japanese ethnicity (the latter being an increased risk factor for gefitinib-associated interstitial lung disease).

Participants were treated with daily oral gefitinib 250 mg for 7 days in addition to 50 mg/m^2^ of i.m. methotrexate on Day 1. Quantitative serum hCG measurement was repeated on Day 4 and Day 7 of treatment, and initial treatment success was defined as a ≥15% fall in serum hCG between these two measurements. Additional doses of methotrexate at 50 mg/m^2^ were administered where this did not occur, or where there was a significant rise in the serum hCG between Day 1 and Day 4. Serum hCG was then measured weekly until there was complete resolution of the EP, defined as a serum hCG of ≤15 IU/l. Haematological, renal and hepatic blood indices were monitored at each visit.

Treatment outcome parameters recorded included time to resolution (days from first methotrexate dose until serum hCG <15 IU/l), safety, tolerability and complication rates. Side effects and symptoms were classified according to the Common Terminology and Criteria for Adverse Events (CTCAE) version 4.03 (National Cancer Institute, National Institutes of Health, U.S. Department of Health and Human Services, June 14, 2010). Participants were contacted at 3, 6 and 12 months post-treatment to document return of menstrual cycles and any subsequent fertility outcomes.

## Results

We recruited eight women with stable non-tubal EPs: five women with interstitial EPs and three women with Caesarean section scar EPs. The range of pretreatment serum hCG levels of participants was between 2458 and 48550 IU/l.

All women were successfully treated with combination gefitinib and methotrexate—none of the women required surgical and/or invasive intervention to achieve cure and furthermore, none of the participants experienced blood loss requiring transfusion. A second dose of i.m. methotrexate was administered to 5/8 of participants, in 3/5 participants because of a significant rise in serum hCG between Days 1 and 4 of treatment and in the remaining 2/5, because the serum hCG had not fallen ≤15% between Days 4 and 7 of treatment. Duration of follow-up ranged from 25 to 196 days. Table I summarizes the non-tubal pregnancy characteristics, treatment and outcomes of each participant. Supplementary Table I provides participant baseline demographic data and additional ultrasound characteristics of the non-tubal pregnancies.

**Table I:** Participant ectopic pregnancy (EP) and treatment details.

Participant	EP type	Day 1 hCG (IU/l)	Day 4 hCG (IU/l)	Day 7 hCG (IU/l)	Fetal heart on ultrasound?	2nd dose of MTX given?	Time to resolution^a^ (days)
1	Interstitial	2458	2049	2350	No	Yes (Day 7)	31
2	Interstitial	6528	6163	6502	Yes	Yes (Day 7)	38
3	Caesarean scar	8716	13 836	9906	No	Yes (Day 4)	48
4	Interstitial	8575	6125	4810	No	No	67
5	Caesarean scar	48 558	54 747	47 551	Yes	Yes (Day 4)	196
6	Interstitial	9730	11 966	12 484	No	Yes (Day 4)	63
7	Interstitial	2649	3662	3497	No	No	25
8	Caesarean scar	8707	5981	3041	No	No	53

MTX, methotrexate.

^a^Resolution defined as serum hCG <15 IU/l.

**Figure 1:**
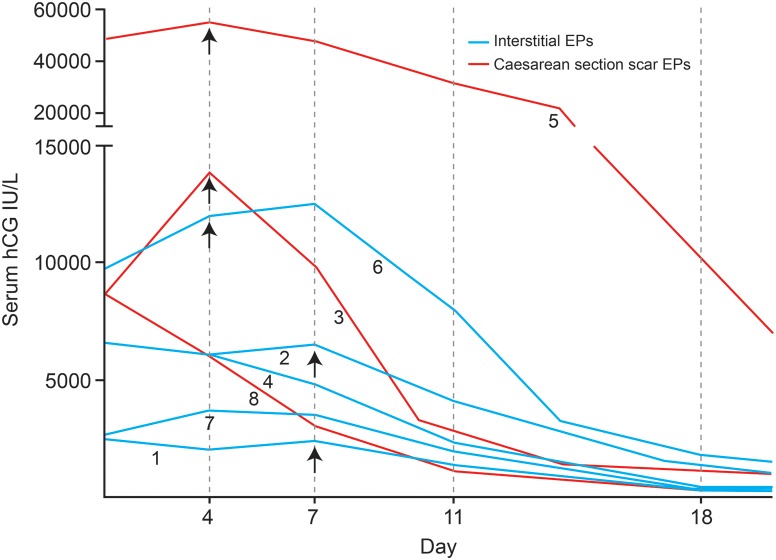
The serum hCG courses of participants. Interstitial ectopic pregnancies shown in blue and Caesarean section scar ectopic pregnancies shown in red. Serum hCGs were measured on Days 4, 7 and 11 of treatment and weekly thereafter until resolution (hCG,15 IU/l), with the protocol commencing again after a repeat dose of methotrexate on Day 4 or Day 7 in 5/8 participants. Numbers on the diagonal correlate to the participant and arrows indicate additional doses of methotrexate.

The hCG courses of the five interstitial EPs were quite varied (Fig. 1). Two participants demonstrated an adequate fall in hCG between Day 4 and Day 7 (participants 4 and 7; see Table I). Participants 1 and 2 experienced an initial fall in serum hCG between Day 1 and Day 4, but this curiously rose to pretreatment levels at Day 7 in both cases. This was despite ultrasound evidence of treatment efficacy in participant 2, where a fetal heart seen pretreatment was not detected on re-scanning at Day 4. In contrast, the hCG courses of the Caesarean section scar EPs all demonstrated a fall in hCG between Days 4 and 7 of treatment, including one with an extremely high pretreatment hCG level of 48 558 IU/l and a fetal heart seen on ultrasound prior to treatment.

**Figure 2:**
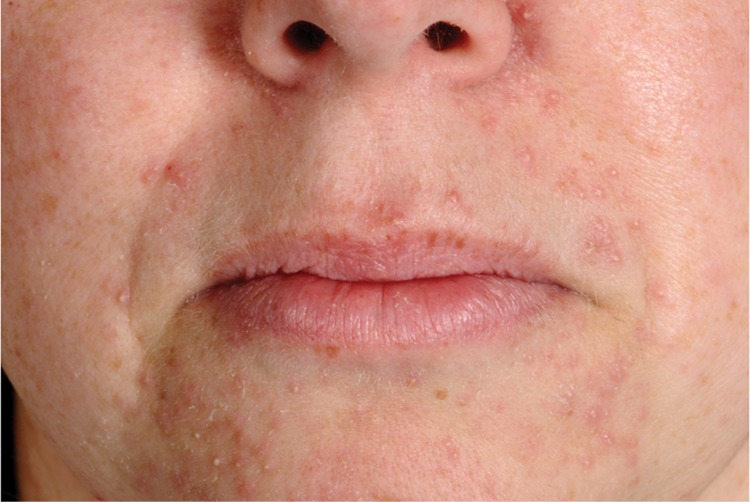
An example of the papulopustular (acneiform) rash experienced by some participants in response to treatment with oral gefitinib. The rash is most prominent in areas exposed to UV light, i.e. the face, neck and décolletage.

The combination of oral gefitinib and systemic methotrexate was well tolerated. The most commonly reported adverse events were a papulopustular rash (Fig. 2), diarrhoea and dizziness, consistent with the known side effect profile of gefitinib. All adverse events were classified as either grade 1 (mild) or grade 2 (moderate) according to the CTCAE, and all women were able to continue with employment and/or family responsibilities after discharge from hospital and during follow-up. All reported adverse events resolved spontaneously after completion of treatment, with only occasional symptomatic treatment required. Importantly, there were no complications of treatment and in particular, none of the participants experienced haemorrhage requiring blood transfusion.

All participants promptly resumed their menstrual cycles (i.e. within 6 weeks of cure), and 3/8 so far have achieved a subsequent spontaneous intrauterine pregnancy. Two of these pregnancies have resulted in the successful births of a healthy infant at term, with a third woman in the second trimester of an uncomplicated pregnancy.

## Discussion

The results of this case series suggest that combination gefitinib and methotrexate therapy could be a safe and effective treatment for non-tubal ectopic pregnancies. The combination treatment was successful in resolving the pregnancies without recourse to surgery or more invasive medical treatments in all cases. Six of the eight women had serum hCG levels of >5000 IU/l, levels where previous studies would suggest the single-dose methotrexate protocol (which includes a second dose if required) may be less effective (Menon *et al.*, 2007).

Non-tubal EPs are uncommon, and consequently, their optimal management has not been firmly established. Additionally, each type of non-tubal EP presents different management challenges (Chetty and Elson, 2009). Advances in ultrasound have enabled earlier and more accurate diagnosis, and the use of minimally invasive techniques has significantly improved the outcomes of women diagnosed with non-tubal EPs (Chetty and Elson, 2009). Nevertheless, non-invasive management remains key to minimizing any risk to subsequent pregnancies.

Non-invasive treatment of non-tubal and indeed all EPs is almost exclusively limited to systemic methotrexate (Hajenius *et al.*, 2000). The effectiveness of methotrexate in the treatment of EP is limited by the pretreatment serum hCG, with ectopic pregnancies with levels >5000 IU/l significantly less likely to be treated successfully (Menon *et al.*, 2007). Because of difficulty accurately characterizing their location with ultrasound, non-tubal EPs are still diagnosed at more advanced gestations with higher pretreatment serum hCG levels, thus limiting the usefulness of this non-invasive approach (Chetty and Elson, 2009). We have demonstrated effective management of women with non-tubal EPs and pretreatment hCGs as high as 48 558 IU/l by combining minimal doses of methotrexate with a short course of gefitinib. This co-treatment approach can achieve better treatment outcomes with lower overall drug exposure (Nilsson *et al.*, 2013).

Another clinical factor negatively associated with methotrexate treatment success is the presence of a fetal heart motion on ultrasound (Bachman and Barnhart, 2012). In this case series of eight women, we successfully treated two non-tubal EPs with embryonic cardiac activity, including one participant who at repeat scanning on Day 4 of treatment, showed the fetal heart motion to have already resolved. These cases provide further encouraging preliminary evidence that this combination treatment is efficacious.

The main goal of non-invasive treatment is preservation of reproductive potential. Importantly, all participants promptly resumed their menstrual cycles after resolution of their non-tubal EPs with combination gefitinib and methotrexate (within 6 weeks). Three participants have subsequently conceived spontaneous intrauterine pregnancies, with two women delivering a healthy infant at term (both normal vaginal deliveries) and a third woman being in her second trimester of an uncomplicated pregnancy.

Our continued experience with combination gefitinib and methotrexate treatment of women with ectopic pregnancies is that it appears safe and well tolerated. There were no serious adverse events recorded during the treatment of these eight women. Non-serious adverse events, predominantly gastrointestinal and mucocutaneous, were consistent with the known side effect profiles of both gefitinib and methotrexate. Furthermore, all side effects were transient, requiring only occasional symptomatic management and completely resolving after discontinuation of treatment.

Treatment of non-small cell lung cancer with gefitinib is associated with interstitial lung disease (ILD) in ∼1% of white and 5% of Japanese patients, and is fatal in up to one-third of cases (Cataldo *et al.*, 2011). We have had no occurrences of ILD in any of our participants from this and other studies, cumulatively 70 women treated with ectopic pregnancies and persistent gestational trophoblastic disease from published and unpublished data (Skubisz *et al.*, 2013). We have screened for and excluded women with significant pulmonary comorbidities and Japanese ethnicity, and in addition to a short and limited 7-day course of gefitinib, believe to risk of ILD in women of reproductive age to be unlikely.

In conclusion, we believe that combination gefitinib and methotrexate is a promising new treatment approach for non-tubal EPs. Whilst we understand that it requires assessment of efficacy in a large clinical trial before it can be introduced into clinical practice, we believe that it has the potential to reduce the need for surgical intervention, improve future reproductive outcomes and minimize the burden of treatment to both health services and women.

## Supplementary data

Supplementary data are available at http://humrep.oxfordjournals.org/.

## Authors' roles

A.W.H., M.M.S., W.C.D., E.M.W., P.N. and S.T. recruited and treated the participants. A.W.H, M.M.S, S.T. and T.G.J. collated and analysed the data. A.W.H., M.M.S. and S.T. drafted the manuscript. E.M.W. and T.G.J. provided clinical and intellectual oversight. All authors critically reviewed the manuscript and approved the final version.

## Funding

This work was supported by National Health and Medical Research Council of Australia (NHMRC) Project Grants #606611 (S.T. and T.G.J.), #1008276 (S.T., E.M.W. and T.G.J.); NHMRC Career Development Fellowship #1050765 (S.T.); The Monash Institute of Medical Research Flagship Grant (S.T., T.G.J., E.M.W.), The Helen MacPherson Trust (ST), The Victorian Government's Operational Infrastructure Support Program (T.G.J., E.M.W.) and an MRC Clinician Scientist Fellowship and MRC Centenary Award (G0802808, A.W.H.). Funding to pay the Open Access publication charges for this article was provided by the University of Edinburgh.

## Conflict of interest

T.G.J., and S.T. are joint holders of patents that relate to the use of EGFR inhibition in treating ectopic pregnancies.

## Supplementary Material

Supplementary Data
