# Supplementary material for: Combination gefitinib and methotrexate treatment for non-tubal ectopic pregnancies: a case series
Source: Hum Reprod. 2014 May 7;29(7):1375–9. doi: 10.1093/humrep/deu091 (PMC4059335; doi:10.1093/humrep/deu091)
Supplement: Supplementary Data [file supp_29_7_1375__index.html]

Combination gefitinib and methotrexate treatment for non-tubal ectopic pregnancies: a case series — Supplementary Data 

# Combination gefitinib and methotrexate treatment for non-tubal ectopic pregnancies: a case series

## Supplementary Data

Supplementary Data

**Files in this Data Supplement:**

- Supplementary Data - pdf file
