# Supplementary material for: Combination gefitinib and methotrexate treatment for non-tubal ectopic pregnancies: a case series
Source: Hum Reprod. 2014 May 7;29(7):1375–9. doi: 10.1093/humrep/deu091 (PMC4059335; doi:10.1093/humrep/deu091)
Supplement: Supplementary Data [file supp_deu091_deu091_suppl_table1.pdf]

Human Reproduction, Vol.0, No.0 , 2014
doi:10.1093/humrep/deu091

human reproduction

SUPPLEMENTARY DATA

Supplementary Table I Participant baseline demographic and additional ultrasound details.

Participant	1	2	3	4	5	6	7	8
Age	30	22	33	23	43	31	33	35
Parity	0	1	2	0	2	0	1	2
BMI (kg/m^2)	23	26.2	31.2	38	30	27.8	25	29
Previous EP	No	No	No	No	No	No	No	No
Smoker	Ex	No	Yes	Ex	No	No	No	Ex
EP location	Interstitial	Interstitial	CS scar	Interstitial	CS scar	Interstitial	Interstitial	CS scar
GS size (mm)	6 × 5 × 7	19 × 5 × 14	12 × 4 × 9	10	32 × 18 × 20	13 × 15 × 12	11 × 7 × 7	13 × 15 × 16
Pelvic free fluid?	No	Small	No	No	No	No	No	Trace
Desiring fertility?	Yes	Yes	No	Yes	No	Yes	Yes	Yes
Subsequent pregnancy	Live birth	Live birth	Nil	EDD12/04/14	Nil	Nil	EDD22/07/14	Nil

EDD, estimated date of delivery; CS, Caesarean section.
